# Cre-loxP Reporter Mouse Reveals Stochastic Activity of the *Foxp3* Promoter

**DOI:** 10.3389/fimmu.2019.02228

**Published:** 2019-09-20

**Authors:** Peter D. Bittner-Eddy, Lori A. Fischer, Massimo Costalonga

**Affiliations:** Division of Periodontology, Department of Developmental and Surgical Sciences, School of Dentistry, University of Minnesota, Minneapolis, MN, United States

**Keywords:** *Foxp3*, Treg cells, Th17 cells, Cre recombinase, fate-tracking

## Abstract

Mouse models that combine specific loxP-flanked gene sequences with Cre recombinase expressed from cell-regulated promoters have become important tools to investigate gene function. Critically however, expression of Cre recombinase may not always be restricted to the target cell or tissue of interest due to promiscuous activity of the driving promoter. Expression of Cre recombinase and, by extension, excision of the loxP-flanked gene may occur in non-target cells and may not be readily apparent. Here we report on the fidelity of Cre recombinase expressed from the *il17a* or *Foxp3* promoters by combining them with a constitutively expressed floxed-stopped tdTomato reporter gene. *Foxp3*-driven Cre recombinase in F_1_ mice induced tdTomato red fluorescent protein in Treg cells but also in a range of other immune cells. Frequency of tdTomato expression was variable but positively correlated (*p* < 0.0001) amongst lymphoid (B cells and CD8 T cells) and blood-resident myeloid cells (dendritic cells, monocytes, neutrophils) suggesting stochastic activity of the *Foxp3* promoter rather than developmental regulation in common ancestral progenitors. Interestingly, frequency of tdTomato^+^ dendritic cells, monocytes and neutrophils did not correlate with the tdTomato^+^ fraction in eosinophils, indicating that activity of the *Foxp3* promoter in eosinophils occurred after the split from a common multipotent progenitor. When these F_1_ mice were crossed to achieve homozygosity of the promoter and reporter gene, a novel visually red phenotype was observed segregating amongst littermates. The red coloration was widespread and prevalent in non-immune tissues. Thymocytes examined from these red mice showed that all four subsets of immature thymocytes (CD4^−^ CD8^−^) based on differential expression of CD25 and CD44 were expressing tdTomato. Finally, we show evidence of *Foxp3* Cre recombinase independent tdTomato expression, suggesting germ line transmission of an activated tdTomato reporter gene. Our data highlights potential issues with conclusions drawn from using specifically the B6.129(Cg)-Foxp3^tm4(YFP/Cre)Ayr^/J mice.

## Introduction

The Cre-loxP system is a powerful genetic tool that allows for precise *in vivo* excision of DNA that is flanked by direct repeats of the loxP sequence through action of the Type 1 topoisomerase, Cre recombinase (1–4). The Cre-loxP system is particularly suited to mice and has been widely applied to targeting gene knockouts to specific tissues and cell types. Deletion of engineered loxP-flanked (floxed) gene sequences can be restricted to specific cells of interest through the use of mouse strains that express Cre recombinase from promoters only active in the target cell type. With the development of mice lines that carry constitutively expressed floxed-stopped reporter genes came the opportunity to characterize promoter activity in diverse tissue types and to allow specific immune cells to be tracked and temporal phenotypic changes to be monitored (5–8).

We are interested in examining plasticity in Th17 and Treg cells in the context of a mouse model of periodontitis, an alveolar bone destructive inflammatory disease. The model tests how chronic, long-term exposure to a pathogen associated with periodontitis in humans, reprograms cytokine production in Th17 cells. Plasticity of Th17 cells is well-documented (6, 9–11), and a late developmental switch to IFN-γ expression in Th17 cells has been implicated in the pathologies of a number of inflammatory autoimmune diseases (12–15). Similarly, Treg cells can also switch to IFN-γ-producing Th1-like cells (16) and even IL-17A-producing Th17-like cells (17) under certain inflammatory conditions [reviewed in (18, 19)]. Mouse reporter strains to track temporal changes in cytokine expression in Th17 and Treg cells were generated by crossing a well-characterized transgenic mouse that carries a constitutively expressed floxed-stopped tdTomato reporter gene (5) to mice expressing Cre recombinase under the control of either the *il17a* (6) or *Foxp3* promoters (20), respectively. The mouse line expressing Cre recombinase from the *il17a* promoter has been used to investigate the requirement for transcription factors Tbet and RORγt in Th17 immunopathologies (21), factors required for Th17 differentiation (22, 23) and plasticity in a number of different disease contexts (7, 24, 25). The *Foxp3* Cre recombinase mouse strain has been used to examine the role of IL-10 and IL-27R in Treg function (20, 26). It has also been used to examine the role of signaling molecules STAT3 and TRAF6, and transcriptional factor and regulator Tbet and Blimp1, respectively, in differentiation or plasticity in Treg cells (27–30).

A detailed analysis of mice strains engineered to express Cre recombinase from different promoters active in differentiated myeloid derived immune cells has revealed instances of promiscuous and unexpected promoter activity (31). Here, we present evidence that expands on an earlier report of promiscuous *Foxp3* promoter activity (32). By breeding the *Foxp3* promoter Cre recombinase mouse line to the floxed-stopped tdTomato reporter strain we not only found tdTomato expressed in target Treg cells, but also in other immune cells as well as non-immune cells. Our data underscores the potentially problematic interpretation of results obtained from *in-vivo* mouse models that exploit the *Foxp3* promoter-Cre recombinase murine line. This problem may be exacerbated particularly when the *in-vivo* model is used to specifically to target abrogation of genes in Foxp3^+^ Treg cells.

## Materials and Methods

### Mouse Strains and Genotyping

All mouse experiments were reviewed and approved by the Institutional Animal Care and Use Committee of the University of Minnesota and performed on sex and age-matched (6–8 week) mice unless specified otherwise. The following mice strains, Il17a^tm1.1(iCre)Stck^/J (6), B6.129(Cg)-Foxp3^tm4(YFP/icre)Ayr^/J (20), Foxp3tm9(EGFP/cre/ERT2)Ayr/J (33), and B6.Cg-Gt(ROSA)26Sor^tm14(CAG−tdTomato)Hze^/J (5) were purchased from Jackson Laboratory (Bar Harbor, ME). F_1_ reporter mouse strain *IL17a*^*Cre*+/−^ *tdTomato*^+/−^ was generated by crossing Il17a^tm1.1(iCre)Stck^/J to B6.Cg-Gt(ROSA)26Sor^tm14(CAG−tdTomato)Hze^/J mice. F_1_ reporter mouse strain *Foxp3*^*YFP*/*Cre*+/−^ *tdTomato*^+/−^ was generated by crossing B6.129(Cg)-Foxp3^tm4(YFP/icre)Ayr^/J to B6.Cg-Gt(ROSA)26Sor^tm14(CAG−tdTomato)Hze^/J mice. In F_1_ reporter mice when either the *il17*a or the *Foxp3* promoter is active, cells, and all subsequent daughter cells will permanently express tdTomato through the action of Cre recombinase. When required F_1_ mice were crossed and genotypes of F_2_ and derived F_3_ mice were determined using DNA obtained from tail snips and PCR protocols tailored to the specific transgene (Jackson Laboratory). To generate a tamoxifen-inducible Cre recombinase mouse we crossed Foxp3tm9 (EGFP/cre/ERT2)Ayr/J to B6.Cg-Gt(ROSA)26Sor^tm14(CAG−tdTomato)Hze^/J. Experimental mice were either *Foxp3*^*GFP*/*Cre*/*ERT*2+/+^ *tdTomato*^+/+^ females or *Foxp3*^*GFP*/*Cre*/*ERT*2+^ *tdTomato*^+/+^ hemizygous males. All mice were housed in microisolator cages under specific-pathogen free conditions with food and water *ad libitum* in compliance with the Association for Assessment and Accreditation of Laboratory Animal Care at the University of Minnesota.

### Mating and Administration of Tamoxifen in *Foxp3^*GFP*/*Cre*/*ERT*2+/+^ tdTomato^+/+^* Females

Sibling 8-week-old female *Foxp3*^*GFP*/*Cre*/*ERT*2+/+^ *tdTomato*^+/+^ mice were placed in a cage with one *Foxp3*^*GFP*/*Cre*/*ERT*2+^ *tdTomato*^+/+^ hemizygous male overnight. The following morning females were checked for the presence of a copulation plug. Presence of a copulation plug was used to indicate embryonic day 0 (E0) of gestation. Successful implantation was verified by monitoring female weight gain. In this strain of female mouse the activation of the *Foxp3* promoter drives the expression of Cre recombinase/Estrogen Receptor 2 (ERT2) fusion protein. Binding of estrogen or estrogen analogs like Tamoxifen to ERT2 lets the Cre/ERT2 complex into the nucleus allowing the endonuclease Cre to excise the flox-stop-flox upstream to the tdTomato which in turns is driven by the very strong ROSA-26 promoter. On E12 and E13 of gestation, 8 mg of Tamoxifen (Sigma-Aldrich, Milwaukee, WI) suspended in 200 μl olive oil (Ward's Science, Rochester, NY) was delivered via oral gavage into the stomach as previously described (33). In these animals, removal of the floxed stop codon and expression of tdTomato red fluorescent protein is controlled temporally by administration of tamoxifen. Tamoxifen is capable of crossing the placental barrier and has been utilized to induce fetal expression of reporter genes (34, 35). However, tamoxifen treatment during pregnancy has been shown to interfere with normal labor and delivery, necessitating the use of fetal mice in this study (35).

### Harvest of Fetal Mouse Tissues

Female mice were euthanized on E20 with CO_2_ followed by cervical dislocation. Afterwards females were disinfected externally with 70% ethanol prior to opening the abdominal cavity. Each uterine chain was rinsed three times with sterile PBS to remove maternal blood contamination prior to separation of fetoplacental units. Each fetoplacental unit was dissected into individual wells of a 12-well plate. After opening of the amniotic sac (fetal membranes), placentas were immediately separated from fetuses and fetuses were rinsed in PBS again to remove any traces of maternal blood contamination as previously described (36). All fetuses were decapitated prior to tissue collection to ensure humane euthanasia. Lungs and spleen were collected from each fetal unit and processed separately.

### Analysis of Cells From Cervical Lymph Nodes and Fetal Lung and Spleen by Flow Cytometry

Single-cell suspensions were isolated from cervical lymph nodes (CLN) and fetal spleens by standard techniques (37). Cells were stained with viability dye Zombie Aqua (BioLegend, San Diego, CA) and Fc receptors blocked using anti CD16/CD32 antibody (eBioscience; clone 93). Cells were next stained with anti-mouse CD3 (BioLegend; clone 17A2), CD4 (eBioscience; clone RM4-5), CD8 (eBioscience; clone 53-6.7) and B220 (eBioscience; clone RA3-6B2) fluorochrome-conjugated mAbs to identify B cells, CD4 T cells and CD8 T cells. Cell phenotypes were sorted on an LSR II flow cytometer (BD Biosciences, San Jose, CA) and fluorescence emissions analyzed with FlowJo software (Tree Star, Ashland, OR). For analysis of lung cells, lungs were minced, treated with 2 mg/ml collagenase D and 1 mg/ml DNAse for 30 min at 37°C followed by a further 10 min with 5 nM EDTA to aid cell dissociation. Minced lung fragments were mashed over nylex membrane, and washed and filtered to produce a single cell suspension. The entire cell suspension was stained with Zombie Aqua, Fc receptors blocked using anti CD16/CD32 antibody then stained with anti-mouse CD45 (BioLegend; clone 30-F11), CD31 (BioLegend; clone 390) and CD326 (BioLegend; clone G8.8) fluorochrome-conjugated mAbs to identify epithelial (CD45^−^, CD326^+^, CD31^−^), endothelial (CD45^−^, CD326^−^, CD31^+^) and immune cells (CD45^+^, CD326^−^, CD31^−^). Stained cells were analyzed as above.

### Identification of Immune Cells in Blood by Flow Cytometry

Blood samples from *Foxp3*^*YFP*/*Cre*+/−^ *tdTomato*^+/−^ and *IL17a*^*Cre*+/−^ *tdTomato*^+/−^ reporter mice were prepared for flow cytometry as previously described (38). Zombie Aqua stained, FC receptor blocked blood cells were stained with anti-mouse CD11b (clone M1/70), F4/80 (clone BM8), Ly-6G (clone 1A8), B220 (clone RA3-6B2), and I-A/I-E (clone M5/114.15.2) fluorochrome conjugated mAbs from BioLegend. Cells were acquired on an LSR II flow cytometer (BD Biosciences, San Jose, CA) and fluorescence emissions analyzed with FlowJo software (Tree Star, Ashland, OR).

### Phenotypic Analysis of Thymocytes

Thymi were dissected from 4-week-old mice and single-cell suspensions stained with Zombie Aqua and FC receptors blocked as described earlier. Thymocytes were stained with anti-mouse CD3, CD4, CD8, CD44 (BioLegend; clone IM7), and CD25 (BioLegend; clone 3C7) flurochrome-conjugated mAbs. Cell phenotypes were sorted on an LSR II flow cytometer and mature and immature thymocyte subsets identified using FlowJo software.

### Confocal Microscopy

Cervical lymph nodes (CLN) were embedded in O.C.T medium (Sakura Finetek, Torrance, CA) and 10 μm frozen cryostat sections cut. Acetone fixed sections were re-hydrated with PBS, blocked with 5% (v/v) rat serum in PBS, and incubated for 30 min at room temperature with 10 μg/mL anti-mouse B220-Alexa Fluor 488 (eBioscience; clone RA3-6B2) mAb in rat serum supplemented PBS. After serial washes with PBS-Tween20 and PBS, DAPI (Life Technologies, Carlsbad, CA) stained sections were imaged at 40X using a LSM 700 Zeiss confocal laser scanning microscope and specific signals quantified using ZEN software (Carl Zeiss Microscopy GmbH, Jena, Germany).

### Statistical Analysis

Data was analyzed and plotted using Prism 7 software (GraphPad Software, San Diego, CA) and displayed as individual data points with means ± SEM. Correlation was determined from computed Pearson correlation coefficients.

## Results

### Cre Expression in *Foxp3^*YFP*/*Cre*+/−^ tdTomato^+/−^* Mice Results in tdTomato Activation in B Cells and CD8 T Cells

**Figure 1:**
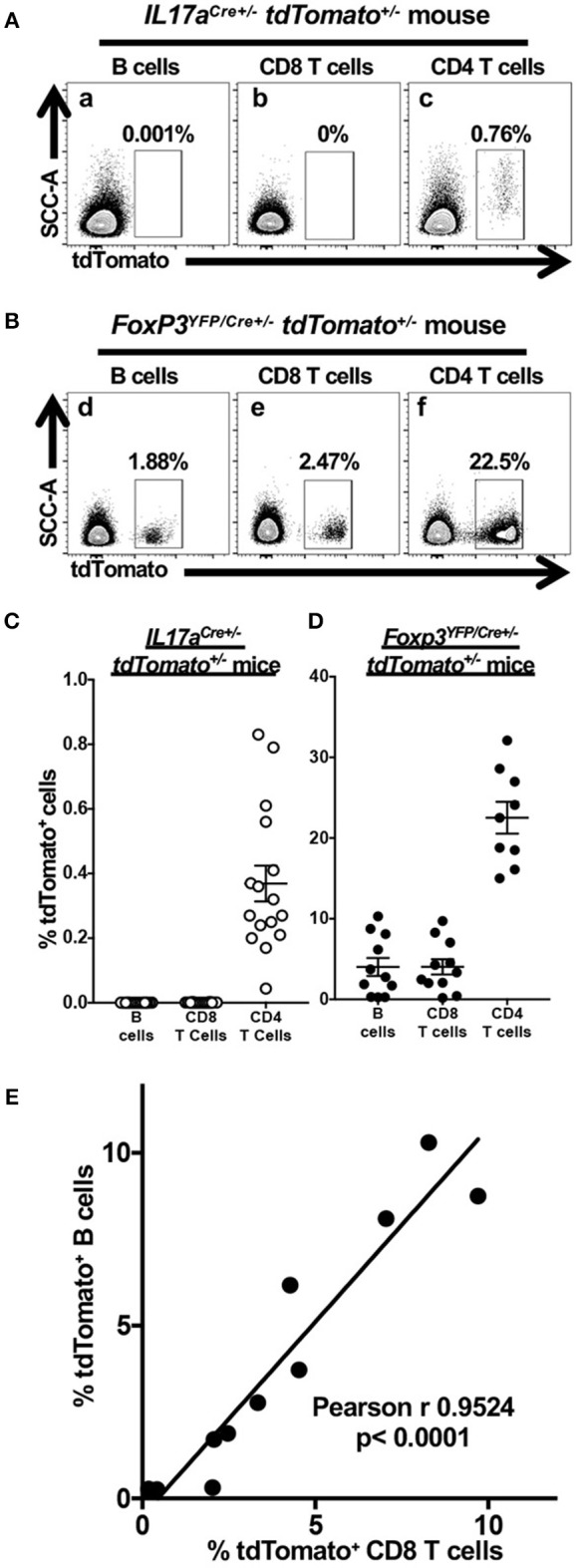
Cre expression in *Foxp3*^*YFP*/*Cre*+/−^ *tdTomato*^+/−^ mice results in tdTomato accumulation in non-target lymphoid cells. Single cell suspensions obtained from CLN of 6–8 week-old *IL17a*^*Cre*+/−^ *tdTomato*^+/−^ or *Foxp3*^*YFP*/*Cre*+/−^ *tdTomato*^+/−^ female mice were stained with Zombie Aqua and incubated with anti-mouse CD3, CD4, CD8, and B220 mAbs to identify live B cells and CD4 and CD8 T cells by flow cytometry. Representative flow cytometry dot plots gated on live (Zombie Aqua^low^) B cells, CD4 T cells or CD8 T cells showing expression of tdTomato in **(A)** *IL17a*^*Cre*+/−^ *tdTomato*^+/−^ or **(B)** *Foxp3*^*YFP*/*Cre*+/−^ *tdTomato*^+/−^ female mice. The percentage of cells falling within a given tdTomato^+^ gate is given. Summary data of tdTomato expression in three lymphoid populations from **(C)** *IL17a*^*Cre*+/−^ *tdTomato*^+/−^ mice or **(D)** *Foxp3*^*YFP*/*Cre*+/−^ *tdTomato*^+/−^. Means and SEMs are plotted along with individual data points. **(E)** The percentage of CD8 T cells expressing tdTomato (x axis) is positively correlated to tdTomato-expressing B cells (y axis) only in *Foxp3*^*YFP*/*Cre*+/−^ *tdTomato*^+/−^ mice. Pearson's correlation test was used to detect the relationship between the two cell frequencies. The regression line is plotted. *N* = 11 mice.

Flow cytometry was used to test the fidelity and robustness of tdTomato expression in cells obtained from the CLN of 6–8 week-old F_1_ female *IL17a*^*Cre*+/−^ *tdTomato*^+/−^ (Figure 1A) or *Foxp3*^*YFP*/*Cre*+/−^ *tdTomato*^+/−^ mice (Figure 1B). To our surprise we found expression of tdTomato in a small but variable fraction of B cells and CD8 T cells in 10 out of the 11 *Foxp3*^*YFP*/*Cre*+/−^ *tdTomato*^+/−^ mice we analyzed (Figures 1Bd,e, respectively). In comparison, expression of tdTomato in B cells and CD8 T cells was effectively zero in all the *IL17a*^*Cre*+/−^ *tdTomato*^+/−^ mice we tested (Figures 1Aa,b, respectively). The percentage of tdTomato expressing CD4 T cells, presumably memory Th17 cells recirculating in CLN of *IL17a*^*Cre*+/−^ *tdTomato*^+/−^ mice (Figure 1C). This Th17 finding is consistent with what observed when staining CD4 T cells for IL-17A in CLN of wild-type mice at homeostasis (37). In contrast, the percentage of tdTomato^+^ CD4 T cells we found in *Foxp3*^*YFP*/*Cre*+/−^ *tdTomato*^+/−^ mice was higher than expected (Figure 1D) based on our observations of Foxp3 expression in CD4 T cells isolated from CLN of similarly aged specific-pathogen free mice (37).

Unexpected expression of tdTomato in B cells and CD8 T cells in *Foxp3*^*YFP*/*Cre*+/−^ *tdTomato*^+/−^ mice prompted us to examine whether the *Foxp3* promoter is independently active in these two lymphoid cell types or it became active in an ancestral lineage progenitor. We found that the percentage of tdTomato^+^ CD8 T cells and B cells is positively correlated in individual *Foxp3*^*YFP*/*Cre*+/−^ *tdTomato*^+/−^ mice (Figure 1E, *p* < 0.0001). A positive correlation suggests that a single event is responsible for the activation of tdTomato expression in these cells and that this event occurred in some, but not all, of the multipotent progenitor cells shared by the B cell and CD8 T cell lineage. Variation in the percentage of cells affected indicates that these events are stochastic in nature rather than developmentally programed.

### tdTomato Expression in Myeloid Cells Isolated From Blood of *Foxp3^*YFP*/*Cre*+/−^ tdTomato^+/−^* Mice Indicates Ancestral *Foxp3* Promoter Activity

**Figure 2:**
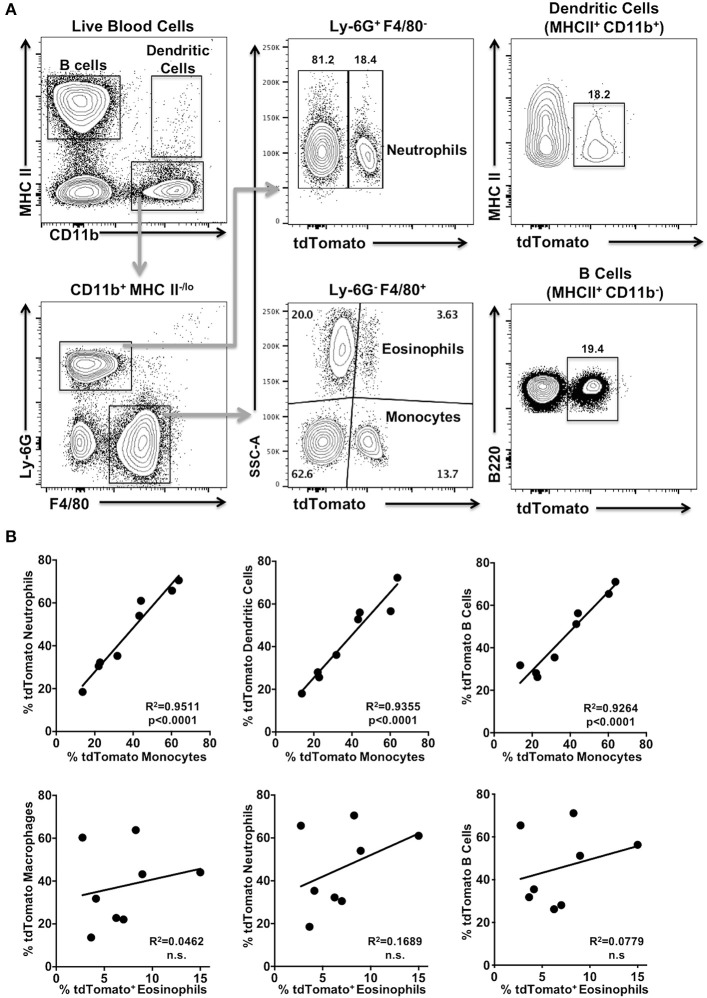
tdTomato is expressed in myeloid cells isolated from blood of *Foxp3*^*YFP*/*Cre*+/−^ *tdTomato*^+/−^ mice. Blood from 6 to 8 week old *Foxp3*^*YFP*/*Cre*−/+^ *tdTomato*^+/−^ female mice was harvested and red blood cells lysed prior to analysis. **(A)** Cells were stained with Zombie Aqua and rat anti-mouse B220, I-A/I-E, CD11b, Ly-6G, and F4/80 mAbs to identify live B cells, monocytes, neutrophils, dendritic cells, and eosinophils by flow cytometry. Representative flow cytometry plots of live cells obtained from a blood sample are shown. Numbers are indicative of the percentage of cells in a given gated population. **(B)** Pearson's correlation test was used to detect the relationship between the frequency of two given tdTomato^+^ cell populations within a mouse. The regression line is plotted and significance given. Not significant (n.s). *N* = 8 mice.

The observation that the tdTomato reporter gene appeared to be activated in multipotent progenitor cells leading to the B and T cell lineages in *Foxp3*^*YFP*/*Cre*+/−^ *tdTomato*^+/−^ mice led us to question if tdTomato expression is also activated in myeloid immune cells. Here we chose to sample blood. We consistently found expression of tdTomato in subpopulations of neutrophils, dendritic cells, eosinophils and monocytes obtained from *Foxp3*^*YFP*/*Cre*+/−^ *tdTomato*^+/−^ mice (Figure 2A), in addition to B cells as expected from our results above. These cells are all non-target cell types. Myeloid cell types in *IL17a*^*Cre*+/−^ *tdTomato*^+/−^ mice did not express tdTomato, although we did observe significant expression of tdTomato in γδ T cells (data not shown), consistent with reported robust expression of IL-17A in these cells (24, 25).

Next we determined if frequencies of tdTomato^+^ cells in these four myeloid populations were correlated with each other and with the tdTomato^+^ B cell fraction in blood samples from *Foxp3*^*YFP*/*Cre*+/−^ *tdTomato*^+/−^ mice. We found significant correlation between the frequency of tdTomato^+^ monocytes and the tdTomato^+^ fraction we detected in both dendritic cells and neutrophils (Figure 2B; *p* < 0.0001), as well as B cells. Interestingly, the frequency of tdTomato^+^ eosinophils was not correlated with the corresponding tdTomato^+^ populations in dendritic cells, neutrophils, monocytes or B cells (Figure 2B; and data not shown).

### YFP Expression in tdTomato^+^ Cells Is Restricted to CD4 T Cells in *Foxp3^*YFP*/*Cre*+/−^ tdTomato^+/−^* Mice

**Figure 3:**
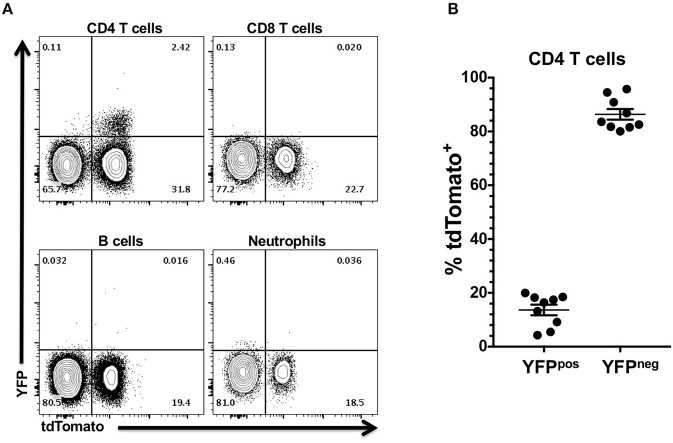
YFP expression in tdTomato^+^ cells from *Foxp3*^*YFP*/*Cre*+/−^ *tdTomato*^+/−^ mice is restricted to the CD4 T cell compartment. CLN and pooled oral mucosa of 6–8 week old *Foxp3*^*YFP*/*Cre*+/−^ *tdTomato*^+/−^ female mice were stained with rat anti-mouse CD3, CD4, CD8, and B220 mAbs (CLN) or rat anti-mouse CD45, I-A/I-E, CD11b, Ly-6G, and F4/80 mAbs (oral mucosa). All cells were stained with Zombie Aqua to discriminate live from dead cells. **(A)** Representative flow cytometry plots showing expression of YFP in CD4 T cell, CD8 T cell, B cell from CLN, and in neutrophils from three pooled oral mucosa samples. Numbers are indicative of the percentage of cells in a given gated population. **(B)** Summary plot data showing percentage of YFP^+^ or YFP^−^ CD4 T cells. Individual data points are shown along with means and SEMs. *N* = 9 mice.

Our data indicated that the *Foxp3* promoter is active or has been active in multipotent progenitor cells other than those leading directly to Treg ontogeny. These results prompted us to determine if the *Foxp3* promoter is currently active in the non-Treg tdTomato^+^ cells. To address this question, we exploited the fact that the *Foxp3* promoter also drives expression of the YFP reporter in *Foxp3*^*YFP*/*Cre*+/−^ *tdTomato*^+/−^ mice. We reasoned that only cells with an active *Foxp3* promoter would also be positive for YFP. YFP expression in CD4 T cells in the CLN and oral mucosa were almost exclusively tdTomato^+^ (Figure 3A). We found no evidence of YFP expression driven by *Foxp3* promoter activity in tdTomato^+^ neutrophils, B cells or CD8 T cells. This data further supports our contention that the *Foxp3* promoter is only transiently active in multipotent progenitor cells that lead to neutrophils, B cells and CD8 T cells and other immune cells. Interestingly, the majority of tdTomato^+^ CD4 T cells were no longer expressing YFP (Figure 3B). Since all mice were kept in specific pathogen-free conditions, it is highly unlikely that all tdTomato^+^ CD4 T cells are former Treg cells that have ceased *Foxp3* promoter activity. Presumably, these tdTomato^+^ CD4 T cells derive from lymphoid progenitors that have transiently activated the *Foxp3* promoter, expressed Cre recombinase and in doing so have activated the tdTomato reporter.

### Stochastic Activity of *Foxp3* Promoter Revealed by Variable Expression of tdTomato in CD4 T Cells Isolated From Spleens of Fetal *Foxp3^*GFP*/*Cre*/*ERT*2+/+^ tdTomato^+/+^* Mice

**Figure 4:**
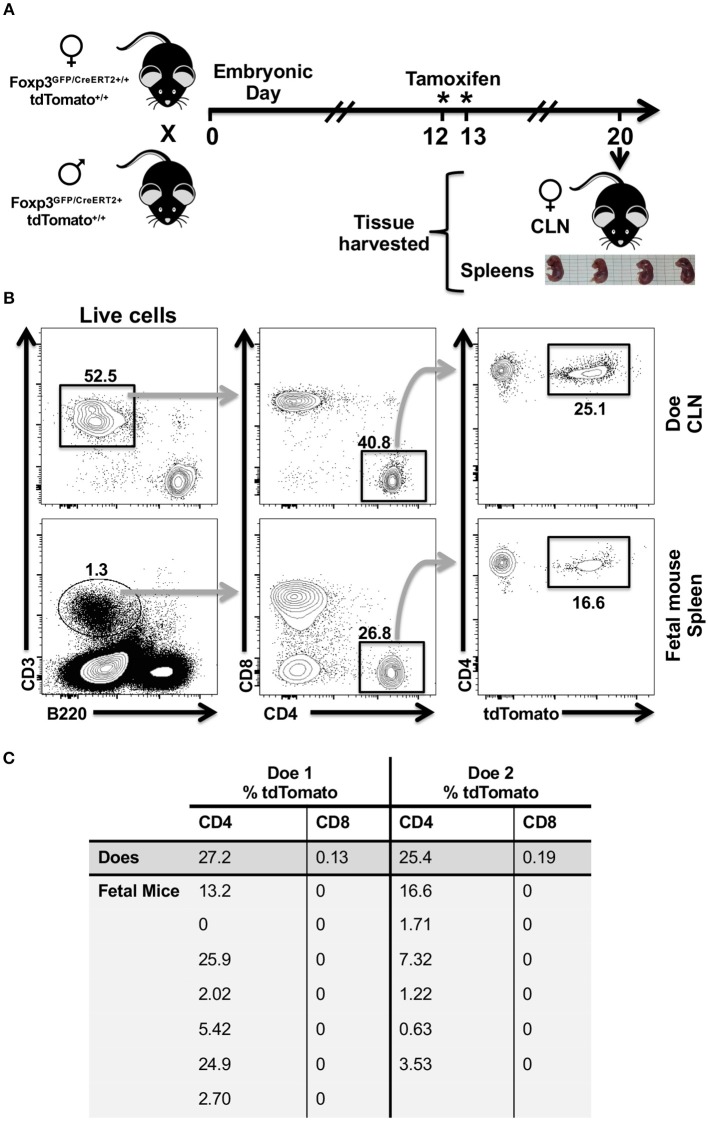
CD4 T cells recovered from the spleens of fetal mice cohorts show variability in tdTomato^+^ frequency in a tamoxifen-inducible Foxp3-Cre model. **(A)** Cartoon showing experimental timeline and design. Eight-week old sibling female *Foxp3*^*GFP*/*Cre*/*ERT*2+/+^ *tdTomato*^+/+^ mice were mated to the same hemizygous *Foxp3*^*GFP*/*Cre*/*ERT*2+^ *tdTomato*^+/+^ male mouse. Females (Does) that had evidence of a copulation plug on embryonic day 0 and that had gained weight by E12 compared to copulation plug minus siblings were orally administered tamoxifen on E12 and E13. **(B)** Single cell suspensions were obtained from CLN of does and fetal mice spleens, stained with Zombie Aqua and rat anti-mouse mAbs to identify CD4 and CD8 T cells by flow cytometry. Representative flow cytometry plots of CLN and a single fetal mouse spleen are shown. Numbers indicate the percentage of cells within a given gate. **(C)** Summary table showing percentage of tdTomato^+^ CD4 and CD8 T cells identified in the spleens of fetal mice and in the CLN of does. ^*^Day of tamioxifen administration.

*Foxp3*^*GFP*/*Cre*/*ERT*2+/+^ *tdTomato*^+/+^ mouse allows activation of tdTomato in cells that only have an active *Foxp3* promoter at the time of tamoxifen exposure, thereby avoiding the confounding effect of transient and historical *Foxp3* activity in multipotent progenitor cells. Moreover, this reporter mouse allows us to test for temporal stochastic activity of *Foxp3* in a range of tissues. Tamoxifen readily crosses the placenta. However, preliminary experiments showed that tamoxifen administrated to pregnant females prevented term birth. To circumvent this issue, we analyzed tdTomato expression in tissues of fetal mice. Published expression data indicated that the *Foxp3* gene may be active by embryonic day 12 (E12). Foxp3 expression in the central nervous system was been reported on E11.5 through E18, and well as the E14.5 limb and liver (39, 40). Expression in the hemolymphoid system, particularly the developing thymus, as well as alimentary system by E15.5 has been reported (41). Other groups however, have reported that *Foxp3* activity at E15.5 only in the reproductive tract (42). This may speak either to the variable nature of Foxp3 expression or differences in analysis. Given these premises, we opted to administer tamoxifen to two pregnant sibling females at E12 and E13 and then harvested lungs and spleens from E20 fetal mice and CLN from the does (Figure 4A). Given that the half-life of Tamoxifen is 5–7 days, this would introduce a sufficient concentration into the systemic circulation to induce red fluorescent protein expression at E14.5-15.5 in the fetus. Foxp3 protein has not been detected in fetal mouse lungs at E14.5, so any red expression in the lung should be limited to transiting immune cells (43). In lung harvested from fetal mice, we found no evidence of tdTomato expression in the epithelial (CD45^−^, CD326^+^, CD31^−^) or endothelial compartments (CD45^−^, CD326^+^, CD31^−^) nor in the relatively abundant CD45^+^ cell fraction (data not shown). However, we observed expression of tdTomato in some CD4 T cells isolated from the fetal spleen and from the CLN of each doe (Figure 4B). The frequency of tdTomato^+^ CD4 T cells was highly variable within fetal cohorts ranging from 0 to 25.9% in one cohort and 0.63 to 16.6% in the other, but was very similar in the cervical lymph node samples from the two sibling does (Figure 4C). We also observed a small population of cervical lymph node-resident tdTomato^+^ CD8 T cells in the does consistent with CD8 Treg cells (44–48), but these cells were absent in all fetal spleens (Figure 4C). tdTomato^+^ expression was also absent within the larger B cell population of all fetal spleens (data not shown).

### Widespread Expression of tdTomato in Homozygous *Foxp3^*YFP*/*Cre*+/+^ tdTomato^+/+^* Mice

**Figure 5:**
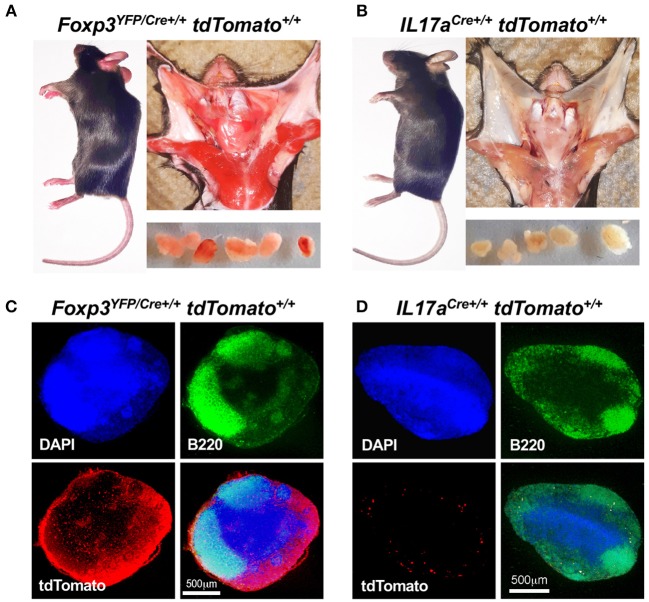
*Foxp3*^*YFP*/*Cre*+/+^ *tdTomato*^+/+^ mice express tdTomato in non-immune tissues. Visual comparison of *Foxp3*^*YFP*/*Cre*+/+^ *tdTomato*^+/+^ **(A)** and *IL17a*^*Cre*+/+^ *tdTomato*^+/+^ mice **(B)** showing “red” coloration in tissues of the *Foxp3*^*YFP*/*Cre*+/+^ *tdTomato*^+/+^ mouse. Dissected CLN are shown in the lower panels of each montage. Frozen sections from CLN of *Foxp3*^*YFP*/*Cre*+/+^ *tdTomato*^+/+^ **(C)** and *IL17a*^*Cre*+/+^ *tdTomato*^+/+^ mice **(D)** were stained with DAPI and rat anti-mouse B220 mAb conjugated to FITC and images captured at 40 X using a confocal microscope. Red fluorescence was captured in the tdTomato channel. Merged images, with a scale bar (500 μm), are shown in the lower right quadrant of each photograph.

During the process of generating homozygous mouse lines, we observed that crosses involving F_1_ *Foxp3*^*YFP*/*Cre*+/−^ *tdTomato*^+/−^ mice generated a novel “red” phenotype that was clearly segregating amongst F_2_ littermates. This phenotype was not observed in our prior F_1_ experimental mice. This red phenotype typically correlated with a double homozygous *Foxp3*^*YFP*/*Cre*+/+^ *tdTomato*^+/+^ genotype in female pups or a *Foxp3*^*YFP*/*Cre*+^ *tdTomato*^+/+^ genotype in male pups. Note here that the *Foxp3* transgene is X-linked. This red phenotype was not seen in any of the double homozygous *IL17a*^*Cre*+/+^ *tdTomato*^+/+^ pups. The appearance of this red coloration was striking and seemed widespread in non-immune tissue. To examine this further we dissected female mice that were genotypically *Foxp3*^*YFP*/*Cre*+/+^ *tdTomato*^+/+^ (Figure 5A) or *IL17a*^*Cre*+/+^ *tdTomato*^+/+^ (Figure 5B). In addition to skin cells, the red coloration was clearly visible in cells that comprise muscle, salivary glands and the capsule of CLN suggesting widespread expression of tdTomato in non-immune cells. We reasoned that a cervical lymph node would give a clear picture of tdTomato expression in immune cells as well as non-immune cells that comprise the capsule, cortex and medullary stroma. Using confocal microscopy CLN sections stained with DAPI and anti-B220, it was obvious that tdTomato is strongly expressed in the capsule and T cell zones and to a lesser extent in the B cell zone of CLN obtained from a red mouse (Figure 5C). In contrast, only a few tdTomato^+^ cells were seen in lymph nodes taken from an *IL17a*^*Cre*+/+^ *tdTomato*^+/+^ mouse (Figure 5D). These cells are presumably Th17 cells and were primarily located along the fringes of the B cell zone.

### Activation of the *Foxp3* Promoter Occurs Prior to Thymocyte Selection in Red *Foxp3^*YFP*/*Cre*+/+^ tdTomato^+/+^* Female Mice

**Figure 6:**
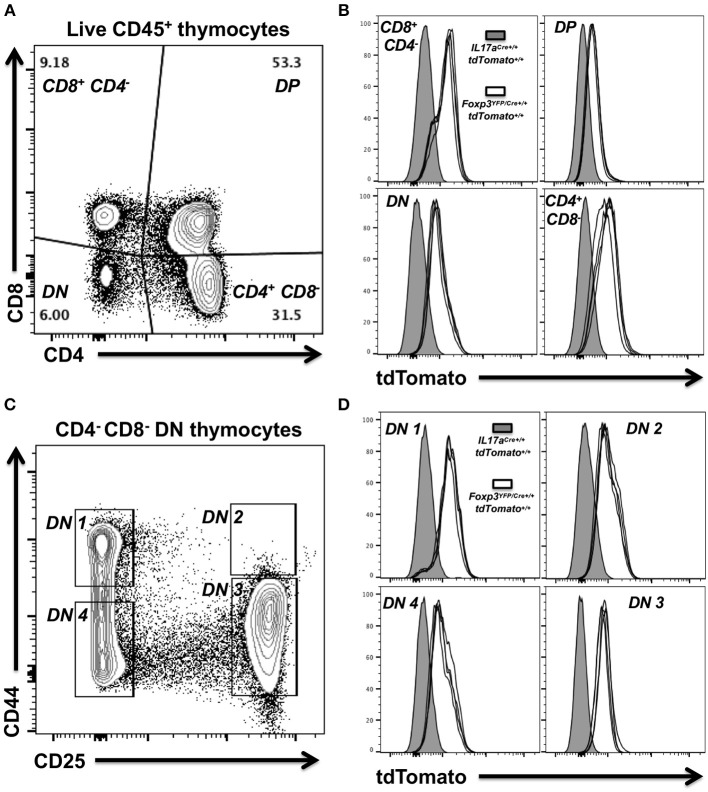
Expression of tdTomato occurs early during thymocyte selection in *Foxp3*^*YFP*/*Cre*+/+^ *dtTomato*^+/+^ homozygous female mice. Thymocytes were prepared from thymi isolated from 4 week-old female *Foxp3*^*YFP*/*Cre*+/+^ *tdTomato*^+/+^or *IL17a*^*Cre*+/+^ *tdTomato*^+/+^ mice. Live mature and immature thymocytes were identified using Zombie Aqua, stained with a panel of rat anti-mouse mAbs and analyzed by flow cytometry. **(A)** Representative flow cytometry plot showing gating strategy used to identify classic immature double negative (DN—CD4^−^ CD8^−^) or double positive (DP—CD4^+^ CD8^+^) thymocytes and mature CD4 and CD8 T cells. Numbers shown indicate the percentage of cells in a given gated population. **(B)** Summary plot data from four female *Foxp3*^*YFP*/*Cre*+/+^ *tdTomato*^+/+^ mice showing tdTomato expression in the four thymocyte populations identified. Thymocytes obtained from a single 4 week-old female *IL17a*^*Cre*+/+^ *tdTomato*^+/+^ mouse were used as baseline of no tdTomato. **(C)** Representative flow cytometry plot showing gating strategy used to identify DN1 (CD25^−^ CD44^+^), DN2 (CD25^+^ CD44^+^), DN3 (CD25^+^ CD44^−^), or DN4 (CD25^−^ CD44^−^) thymocyte progenitor populations amongst DN cells (CD4^−^ CD8^−^). **(D)** Summary plot data from four female *Foxp3*^*YFP*/*Cre*+/+^ *tdTomato*^+/+^ mice and the *IL17a*^*Cre*+/+^ *tdTomato*^+/+^ control.

Widespread expression of tdTomato in red *Foxp3*^*YFP*/*Cre*+/+^ *tdTomato*^+/+^ mice together with our data that suggested transient activity of the *Foxp3* promoter in immune progenitor cells, prompted us to examine thymocytes for expression of tdTomato. Thymocytes were prepared from the thymi of four red 4-week-old female *Foxp3*^*YFP*/*Cre*+/+^ *tdTomato*^+/+^ mice and a single age-matched *IL17a*^*Cre*+/+^ *tdTomato*^+/+^ female mouse to serve as a control. Live mature and immature thymocytes were identified by flow cytometry. Classic immature double negative (DN: CD4^−^ CD8^−^) or double positive (DP: CD4^+^ CD8^+^) thymocytes as well as mature CD4^+^ and CD8^+^ thymocytes were identified (Figure 6A). tdTomato expression in each of these thymocyte groups was compared in the equivalent populations obtained from the *IL17a*^*Cre*+/+^ *tdTomato*^+/+^ mouse (gray histogram) and *Foxp3*^*YFP*/*Cre*+/+^ *tdTomato*^+/+^ mice (white histograms; Figure 6B). This analysis clearly showed that tdTomato was expressed in all four of the identified thymocyte populations. DN cells are considered early progenitors, but they themselves can be further subdivided into DN1 (CD25^−^ CD44^+^), DN2 (CD25^+^ CD44^+^), DN3 (CD25^+^ CD44^−^), and DN4 (CD25^−^ CD44^−^) populations based on the expression and sequential loss of cell surface markers CD25 and CD44 (Figure 6C) (49). We decided to also look for tdTomato expression in these thymocyte populations. Our analysis revealed tdTomato expression in the earliest of these progenitor thymocytes, DN4 (CD25^−^ CD44^−^), indicating that activation of the tdTomato reporter gene occurred very early in the stem cell lineage that leads to thymocytes (Figure 6D).

### Expression of tdTomato in *Foxp3^*YFP*/*Cre*−^ tdTomato^+/+^* Male Mice

To determine if the red phenotype is associated with a particular genotype, we used a simple binary visual scoring system to score F_2_ siblings and later generations as red or “non-red” and then used PCR to identify their genotype. As expected from our earlier data, all female mice that were double homozygous *Foxp3*^*YFP*/*cre*+/+^ *tdTomato*^+/+^ had the visual red phenotype (Table 1). While the majority of *Foxp3*^*YFP*/*cre*+^ *tdTomato*^+/+^ males were red, unexpectedly some non-red males of this genotype were also observed. *Foxp3*^*YFP*/*cre*+^ *tdTomato*^+/−^ heterozygous males were equally split between red and non-red phenotypes. In females, the *Foxp3*^*YFP*/*cre*+/+^ *tdTomato*^+/−^ genotype yielded predominantly non-red mice whereas the *Foxp3*^*YFP*/*cre*+/−^ *tdTomato*^+/+^ genotype gave predominantly red mice. Intriguingly, we identified two F_3_ brothers that were visually red that after repeated genotyping showed the presence of only the wild-type *Foxp3* allele and not the *Foxp3*^*YFP*/*cre*^ transgene (data not shown). The mother was *Foxp3*^*YFP*/*cre*+/−^ *tdTomato*^+/+^ and the father was *Foxp3*^*YFP*/*cre*+^ *tdTomato*^+/−^, both having a red phenotype. This result suggests that the tdTomato gene received by these two F_3_ male siblings already had the stop codon excised by activity of *Foxp3*^*YFP*/*cre*^ in germline cell progenitors in one of the parents, thereby making its expression independent of the presence of the *Foxp3*^*YFP*/*cre*^ transgene.

**Table 1:** Genotype distribution associated with “red” phenotype in F_1_, and F_3_, mice.

**Sex**	**Genotype**	**Red**	**Non-red**
Males	*Foxp3^*YFP*/*Cre*+^*tdTomato*^+/+^*	15	2
	*Foxp3^*YFP*/*Cre*+^*tdTomato*^+/−^*	2	2
	*Foxp3^*YFP*/*Cre*−^*tdTomato*^+/−^*	2^*^	8
Females	*Foxp3^*YFP*/*Cre*+/+^*tdTomato*^+/+^*	5	0
	*Foxp3^*YFP*/*Cre*+/+^*tdTomato*^+/−^*	2	8
	*Foxp3^*YFP*/*Cre*+/−^*tdTomato*^+/+^*	7	1

*Mice were sorted into binary “red” and “non-red” groups. Non-red mice were equivalent in coloration of ears, paws, and tails to wild-type C57BU6 mice housed in the same colony. Red mice were as visualized in Figure 5. Mice with an unclear or intermediate red phenotype were discarded and not considered in this analysis. Tail snips were collected for DNA and PCR used to determine genotypes. Male and female genotypes are shown separately as the X-Iinked Foxp3^YFP/Cre^ gene is inherited differently in males and females. Only genotypes that resulted in red mice are listed*.

* *germline transmission independent of Cre recombinase*.

## Discussion

We developed fate-tracking reporter mouse strains to examine plasticity in Th17 and Treg cells in the context of a murine model of chronic periodontitis. Both these cell types have been reported to undergo cytokine reprogramming during conditions of heightened chronic inflammation and a shifting cytokine milieu that develop during a number of CD4 T cell-mediated autoimmune diseases (6, 7, 13, 19, 50, 51). In a preliminary set of experiments to determine the specificity and fitness of the fate-tracking reporter mice, we observed unexpected tdTomato expression in non-target lymphoid and myeloid cells within *Foxp3*^*YFP*/*Cre*+/−^ *tdTomato*^+/−^ mice. Translation of the tdTomato gene construct is tightly controlled in B6.Cg-Gt(ROSA)26Sor^tm14(CAG−tdTomato)Hze^/J mice by an inframe loxP-flanked stop codon (5). Moreover, we did not detect tdTomato in these cells in *IL17a*^*Cre*+/−^ *tdTomato*^+/−^ mice ruling out the possibility that non-target cell expression in *Foxp3*^*YFP*/*Cre*+/−^ *tdTomato*^+/−^ mice is due to leaky translation of the tdTomato transgene. Some CD8 T cells, however, have been reported to act as immuno-regulatory CD8 Treg cells expressing *Foxp3* in a number of murine models of disease (44–48), in bone homeostasis (52) and at low background levels (20). Therefore, detecting expression of tdTomato in CD8 T cells in both the *Foxp3*^*YFP*/*Cre*+/−^ *tdTomato*^+/−^ and *Foxp3*^*GFP*/*Cre*/*ERT*2+/+^ *tdTomato*^+/+^ mice is consistent with these observations, even in homeostatic specific pathogen-free mice. It has been reported that CD8 Treg cells do not express CD28 or have low expression (46, 53) and they appear to differentiate in the thymus of mice (53). We did not determine CD28 expression on tdTomato^+^ CD8 T cells. However, we consider it unlikely that these are CD8 Treg cells as our analysis of B cells and CD8 T cells revealed that expression of tdTomato appeared to be stochastically determined in *Foxp3*^*YFP*/*Cre*+/−^ *tdTomato*^+/−^ mice, ruling out a developmentally regulated program of CD8 Treg cell polarization. Moreover, the frequencies of tdTomato^+^ CD8 T cells in *Foxp3*^*YFP*/*Cre*+/−^ *tdTomato*^+/−^ mice were at least 5–10-fold higher than YFP^+^ Foxp3^+^ CD8 T cells reported in the original mouse strain (20). Furthermore, the frequency of tdTomato^+^ CD8 T cells we observed in the CLN from our *Foxp3*^*GFP*/*Cre*/*ERT*2+/+^ *tdTomato*^+/+^ females, a model that avoids the issue of tdTomato reporter activation due to transient stochastic *Foxp3* driven Cre expression, was consistent with reports of CD8 Treg cell frequency (20).

We chose the B6.129(Cg)-Foxp3^tm4(YFP/Cre)Ayr^/J mouse strain to develop a model whereby we could track the expression of pro-inflammatory cytokines IL-17A and IFN-γ in ex-CD4 Treg cells over the course of a chronic disease using tdTomato expression as a marker of Treg differentiation. However, we found that tdTomato was activated in a broad range of CD4 T cells. Between 80 and 95% of tdTomato^+^ CD4 T cells were not expressing YFP, which by design is the readout of an active *Foxp3* promoter in these mice. We consider it highly unlikely that these YFP negative tdTomato^+^ CD4 T cells could all be exTregs. A more likely scenario is that these cells comprise a combination of naïve or recirculating memory or effector CD4 T cells where transient expression of Cre recombinase has activated the tdTomato reporter gene. This makes such a mouse model highly problematic for tracking authentic ex-Treg cells. For example, in this model it is more likely that YFP negative tdTomato^+^ CD4 T cells expressing IL-17A are simply Th17 cells and not ex-CD4 Treg cells expressing IL-17A de novo (17–19).

tdTomato expression in *Foxp3*^*YFP*/*Cre*+/−^ *tdTomato*^+/−^ mice revealed evidence of the stochastic nature of *Foxp3* activity and that this activity occurred in multipotent progenitor cells of both lymphoid and myeloid lineages. Interestingly, lack of correlation between tdTomato^+^ frequency in the eosinophil population with other myeloid cells examined (i.e., monocytes and neutrophils), suggests that in the eosinophil precursor lineage, *Foxp3* is activated stochastically at a different frequency to other hemopoietic stem cell-derived cells. We speculate that such differences between eosinophils and monocytes, neutrophils, B cells, and dendritic cells may be related to hemopoietic stem cell microenvironments within the bone marrow or fetal liver (54, 55).

Variability in the frequency of tdTomato^+^ cells within populations of CD8 T cells, B cells and monocytes for example is highly suggestive of random *Foxp3* promoter activity outside of the normal developmental program. Our analysis of tdTomato expression in fetal cohorts from *Foxp3*^*GFP*/*Cre*/*ERT*2+/+^ *tdTomato*^+/+^ does underscored this conclusion. If *Foxp3* promoter activity, and therefore Cre recombinase expression, was developmentally regulated then we would expect to see similar tdTomato^+^ frequencies in fetal cohorts given that each fetus was at the same embryonic age when given tamoxifen and when tissues were harvested. The fact that we observed high variation in the frequency of tdTomato^+^ CD4 T cells within a fetal cohort speaks to the random nature of *Foxp3* activity at least in these CD4 T cells. Interestingly, unlike for *Foxp3*^*YFP*/*Cre*+/−^ *tdTomato*^+/−^ mice, in *Foxp3*^*GFP*/*Cre*/*ERT*2+/+^ *tdTomato*^+/+^ mice we did not observe tdTomato expression in CD8 T cells and B cells from spleen or non-immune cells like epithelial and endothelial cells from fetal lung. This observation likely reflects differences in when the *Foxp3* promoter is transiently and/or stochastically activated in embryonic tissue. For example, transient and/or stochastic *Foxp3* promoter activity in multipotent progenitor cells that lead to B and T cell lineages may occur prior to or well-after embryonic day 12 and 13 or when tamoxifen is metabolized.

Both Cre recombinase and the tdTomato transgene construct can be considered dominant traits. Interestingly, we observed a novel striking red phenotype that segregated amongst littermates resulting from F_1_ *Foxp3*^*YFP*/*Cre*+/−^*tdTomato*^+/−^ crosses. This red phenotype primarily was found in mice that carried two copies of each of the transgenes. This novel red phenotype could be attributed to widespread expression of tdTomato beyond the target Treg cell type. In an analysis of immature thymocytes from visually “red” mice, we found that the earliest of the thymocyte progenitors expressed tdTomato suggesting early, albeit transient, expression of the *Foxp3* promoter had occurred even earlier in the lineage. Our data contrasts with an earlier report where B6.129(Cg)-Foxp3^tm4(YFP/Cre)Ayr^/J mouse strain was crossed to *R26*^*RFP*^ or *R26*^*YFP*^ mice (32). These authors did not report issues with expression of RFP or YFP outside of Treg cells, although it appears that homozygous mice were not tested. It is also not clear which *R26*^*RFP*^ reporter mouse they used. Differences between their work and ours may also reflect the differential accessibility of different floxed alleles by Cre recombinase (3, 4). Nonetheless, our work points to potential issues with the interpretation of data generated from crosses involving the B6.129(Cg)-Foxp3^tm4(YFP/Cre)Ayr^/J mouse strain (20, 26–30), particularly if deletion of floxed genes was not monitored in non-target cells and other experimentation, such as adoptive transfer, was not used to confirm the results (27, 56). For example, IL-10 targeted for deletion in Treg cells (20), is produced by a wide range of immune cells and our data suggests that the IL-10 gene could also have been disrupted in those cells, which could have led to a “heightened” loss of IL-10 effect and one that would not be solely attributed to Treg cells. Furthermore, using this mouse strain to target deletion of STAT3 or Tbet in Treg cells would also have likely affected Th17 and Th1 cells, respectively, which calls into question some of the data particularly as it relates to diseases where Th17 or Th1 cells may also play a contributory role (28, 30).

We also reveal one further potential issue of the B6.129(Cg)-Foxp3^tm4(YFP/Cre)Ayr^/J mouse strain. We showed that it was possible for the red phenotype to segregate independently of the *Foxp3*^*YFP*/*Cre*^ transgene. Presumably, excision of the floxed stop signal had occurred early in germline progenitors in one of the parents and that an activated tdTomato gene could now segregate independently of the *Foxp3*^*YFP*/*Cre*^ transgene. It would, therefore, be problematic if negative controls are selected on the basis of the absence of the *Foxp3*^*YFP*/*Cre*^ transgene in littermates that are otherwise homozygous for the floxed target gene. Our data indicates that some of these negative controls may in fact carry the floxed gene deletion and it would be prudent to test for this potential scenario.

In summary, our data shows that the use of the B6.129(Cg)-Foxp3^tm4(YFP/Cre)Ayr^/J mouse strain to generate mutations based on floxed genes targeted to Treg cells requires special consideration of the potential to disrupt these genes in non-target cell types. Additional experimentation would be prudent to confirm results obtained *in vivo* in a whole mouse. Transcription factors, such as Foxp3, may be problematic in forming the basis of a Cre recombinase mouse models, as they are often not tissue or cell specific but have pleiotropic or transient activities. Better models are based on tightly regulated cell specific markers to cell types. The development of the Foxp3 cre-ert2 mouse strain (33), which allows Cre recombinase to be active on nuclear DNA only upon the administration of tamoxifen, would likely mitigate against most of the issues of transient expression from the *Foxp3* promoter that our paper has highlighted.

## Data Availability

The datasets generated for this study are available on request to the corresponding author.

## Ethics Statement

This study was carried out in compliance with the Association for Assessment and Accreditation of Laboratory Animal Care at the University of Minnesota. All mouse experiments were reviewed and approved by the Institutional Animal Care and Use Committee of the University of Minnesota.

## Author Contributions

MC and PB-E designed the experiments and interpreted the data. PB-E drafted the manuscript. All authors performed the experiments and edited and reviewed the manuscript.

### Conflict of Interest Statement

The authors declare that the research was conducted in the absence of any commercial or financial relationships that could be construed as a potential conflict of interest.
